# Insect ATP-Binding Cassette (ABC) Transporters: Roles in Xenobiotic Detoxification and Bt Insecticidal Activity

**DOI:** 10.3390/ijms20112829

**Published:** 2019-06-10

**Authors:** Chao Wu, Swapan Chakrabarty, Minghui Jin, Kaiyu Liu, Yutao Xiao

**Affiliations:** 1 Agricultural Genomics Institute at Shenzhen, Chinese Academy of Agricultural Sciences, Shenzhen 518120, China; wuchao@caas.cn (C.W.); swapan.ag.sau@gmail.com (S.C.); jinminghui722@163.com (M.J.); 2 Institute of Entomology, School of Life Sciences, Central China Normal University, Wuhan 430079, China

**Keywords:** insect, ABC transporters, xenobiotic detoxification, Bt insecticidal activity

## Abstract

ATP-binding cassette (ABC) transporters, a large class of transmembrane proteins, are widely found in organisms and play an important role in the transport of xenobiotics. Insect ABC transporters are involved in insecticide detoxification and *Bacillus thuringiensis* (Bt) toxin perforation. The complete ABC transporter is composed of two hydrophobic transmembrane domains (TMDs) and two nucleotide binding domains (NBDs). Conformational changes that are needed for their action are mediated by ATP hydrolysis. According to the similarity among their sequences and organization of conserved ATP-binding cassette domains, insect ABC transporters have been divided into eight subfamilies (ABCA–ABCH). This review describes the functions and mechanisms of ABC transporters in insecticide detoxification, plant toxic secondary metabolites transport and insecticidal activity of Bt toxin. With improved understanding of the role and mechanisms of ABC transporter in resistance to insecticides and Bt toxins, we can identify valuable target sites for developing new strategies to control pests and manage resistance and achieve green pest control.

## 1. Introduction

ATP-binding cassette (ABC) proteins comprise an extensive and variable transporter superfamily within P-loop motif and are found in all living organisms [1,2,3]. Studies on ABC transporters began in the early 1970s with the biochemical characterization of substrate-binding protein-dependent transport in *Escherichia coli* that was directly energized by hydrolysis of ATP [4,5]. In 1982, cytoplasmic membrane-associated transporter genes in the histidine transport system of *Salmonella typhimurium* (coded by the *hisP* gene) and maltose-maltodextrin transport system of *E. coli* (coded by the *malK* gene) were cloned [6,7]. Concurrently, in mammalian cells, the gene encoding permeability, glycoprotein (P-gp, a large glycosylated membrane protein related to multi-drug resistance) was identified and cloned in 1985 [8,9]. Eventually, substrate-binding transport proteins with ATP-binding subunits were found to constitute a large superfamily of transport proteins and termed ABC transporters in 1990 [10]. On the basis of differences in the ATP-binding sites among insect ABC transporters, the superfamily can be divided into eight subfamilies (ABCA to ABCH) [11].

The primary function of most ABC proteins is ATP-dependent active transport of a broad spectrum of substrates including amino acids, sugars, heavy metal ions and conjugates, peptides, lipids, polysaccharides, xenobiotics and chemotherapeutic drugs across cellular membranes [1,12,13,14], but they are also involved in many other biochemical and physiological processes. In humans, they have also been shown to function as ion channels and receptors [1,12,15]. Because of their ability to transport chemotherapeutic drugs and other hydrophobic substrates such as lipids and hormones, many human ABC superfamily members have been identified as the agent responsible for multidrug-resistance in cancer cells. In fact, the P-glycoprotein ABCB1 (also known as MDR1, multidrug-resistance protein 1), that is overexpressed in multidrug resistant tumor cell lines, was the first ABC protein identified as such [9,16].

Although the ABC transporters have been recognized to be associated with multidrug resistance in humans, bacteria and nematodes, their functional role in arthropods has not been fully studied [17]. Although studies on insect ABC transporters were triggered because they are associated with the evolving field resistance to different Bt toxins and insecticides, the scope of studies on insect ABC transporters has greatly expanded with the advancement of sequencing technology and the annotation of more insect genomes. The availability of insect genome databases provide genomic insights for analyzing the comparative positions and phylogenetic relationships of ABC transporter genes among genetically distant species. The *white* gene in *Drosophila melanogaster* was the first identified insect ABC transporter gene and is involved in the transport of eye pigment precursors [18]. The role of a gene orthologous to *white* has also been confirmed in *Bombyx mori* and *Tribolium castaneum* [19,20]. The upregulation of some ABC transporter genes is associated with resistance to highly effective insecticides such as pyrethroids in some insects [21,22,23]. It is also reported that alterations in the *ABCC* genes are associated with the resistance to Cry toxins from *Bacillus thuringiensis* (Bt) by reducing the binding affinity of Cry toxins to the brush border membrane vesicles in different lepidopteran species [24,25,26]. On the basis of above mentioned evidences, it is noteworthy that ABC transporters have important role in xenobiotic detoxification and Bt-resistance.

Excellent reviews on the role of insect ABC transporters in the transport and resistance to Bt toxin and insecticide have been published previously [14,27,28], so the present review provides an update of our understanding of the evolution, function and role of the ABC transporter superfamily of insects in xenobiotic transport and detoxification.

## 2. Structure and Mechanism of ABC Transporters

Structural models of ABC transporters are based on the crystal structure of different bacterial proteins that act as importers such as vitamin B12 transporter BtuCDF from *E. coli* and exporters such as the multidrug exporter Sav1866 from *Staphylococcus aureus* or related flippases such as MsbA lipid flippase from *E. coli* [29,30,31]. On the basis of their architecture and biochemical activity, the ABC importers have been divided into type I and type II [12,32,33]. The energy coupling factor (ECF) transporters, which differ structurally and functionally from other ABC importers, are sometimes considered as type III ABC importers [34,35,36,37]. However, ABC importers have only been confirmed in prokaryotes, not in eukaryotes [11,15]; therefore, in this review, we focus only on ABC exporters. 

**Figure 1:**
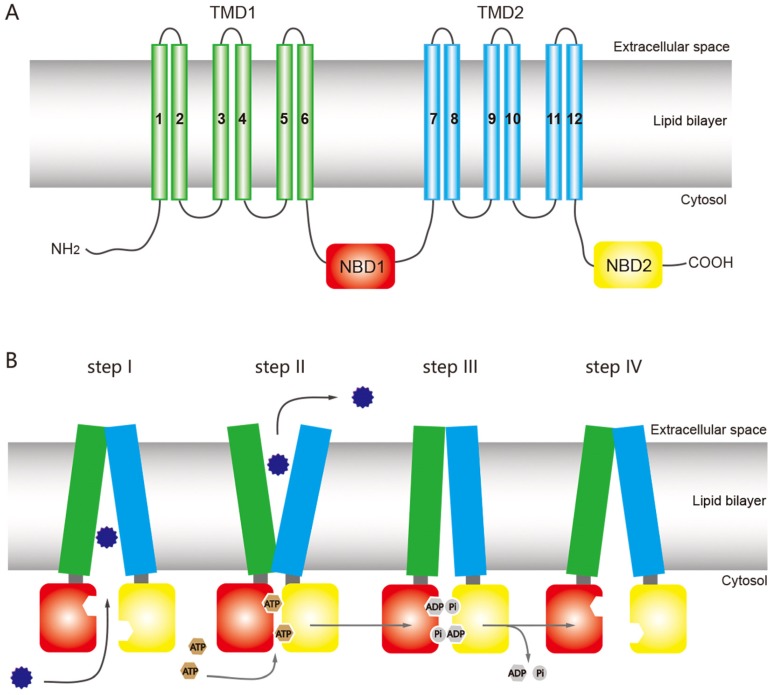
General structure of an ATP-binding cassette (ABC) full transporter (ABC exporter) and the ATP-switch model for the transport mechanism of ABC transporters. (**A**) Typical ABC full transporter with two transmembrane domains (TMDs), TMD1 (green) and TMD2 (sky blue), and two nucleotide-binding domains (NBDs), NBD1 (red) and NBD2 (yellow). Each transmembrane domain (TMD) contains six transmembrane helices. The “long” multidrug-resistance associated proteins (MRPs) of the ABCC subfamily contains an additional TMD (TMD0) at the N terminus [46]. (**B**) The ATP-switch model [14] includes (I) binding of the substrates (12-point blue circle) to the TMDs; (II) subsequent structural changes to the NBDs (red and yellow), hydrolysis of ATP (brown circles), followed by closed dimer formation of the NBDs and major conformational change in the TMDs, which initiates substrate translocation; (III) the ATP is hydrolyzed (gray circles), releasing ADP and Pi, and (IV) finally destabilization of the closed dimer restores its initial open dimer configuration for another new cycle. This figure is drawn by following the previous report of ABC transporter by Dermauw & Van Leeuwen [14].

The structure of ABC transporters is highly conserved among most eukaryotic organisms, including insects. A functional ABC transporter is characterized by the presence of a P-type traffic ATPase, which comprises two cytosolic nucleotide-binding domains (NBDs) and two transmembrane domains (TMDs) [1,12,38] (Figure 1A). The four domains of a functional transporter (2TMDs-2NBDs) are combined in a single polypeptide, forming a full transporter (FT), whereas a half transporter (HT) contains one TMD and one NBD, which are sometimes encoded as separate polypeptides and then fused into multidomain proteins. For ATP binding and hydrolysis, the HT must become a functional transporter by forming homo- or heterodimeric complexes. The NBD contains several highly conserved nucleotide-binding sequences such as the Walker A and B motifs, common in nucleotide-binding proteins (the Walker B motif also provides the catalytic base); d-loop, which contains an aspartate residue and is responsible for forming a salt bridge; Q-loop, which contains a glutamate residue and acts as the attacking nucleophile in ATP hydrolysis; H motif, which has an invariant histidine active site that may be involved in maintaining the stability of the pre-hydrolytic state; and an α-helical region that has the ABC signature sequence (LSGGQ motif) [12,13,39]. The ABC exporter fold, a prominent quaternary structure in TMDs in all ABC exporters, is characterized by 12 transmembrane helices and acts as a switch between different conformational changes and initiates substrate translocation [12]. 

ABC transporters have a common mechanism for exporting substrates across the membrane by hydrolyzing ATP as a pump, but other models have been proposed for the ABC transporter mechanism based on structural and biochemical evidence, including the ATP-switch [39], alternating site [40], constant contact [41,42], and thermodynamic models [43]. Among these models, the ATP-switch model provides a reasonable framework for the transport mechanism [39,44,45] in which repeated communication between NBDs and TMDs occurs in both directions and involves only non-covalent conformational changes. The transport process is initiated by the binding of the substrate to the TMDs, and subsequent structural changes are transmitted to the NBDs, which include ATP-binding and closed dimer formation of the NBDs. Then the closed NBD dimer induces a substantial conformational change in the TMDs. This conformational change initiates translocation of the substrate through a rotation of the TMDs and opening toward the extracellular milieu. Finally, the ATP is hydrolyzed, releasing ADP and Pi and destabilizing the closed dimer conformation to restore its open dimer configuration for another new cycle [39,44,45] (Figure 1B).

## 3. ABC Transporter Subfamilies in Insects

**Figure 2:**
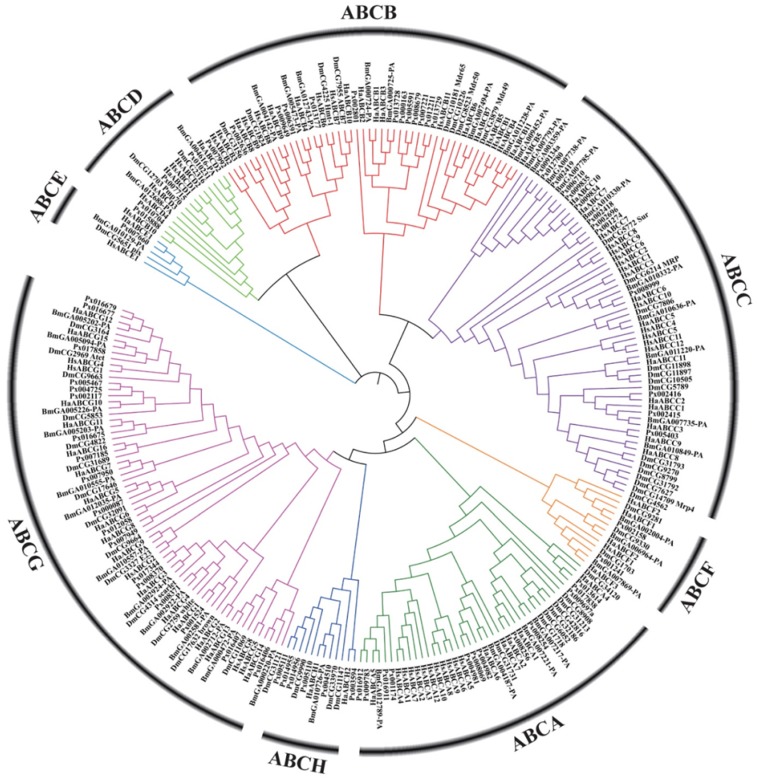
Phylogenetic tree based on amino acid sequences of 262 ABC transporters (Supplementary Material 1) from several insects and humans. The sequences were aligned using MUSCLE. The evolutionary history was inferred using the neighbor-joining method and MEGA-X with 1000 bootstrap replicates. All positions with less than 95% site coverage were eliminated. Species codes: Ha, *Helicoverpa armigera*; Bm, *Bombyx mori*; Px, *Plutella xylostella*; Hs, *Homo sapiens*; Dm, *Drosophila melanogaster*.

In recent years, with the large-scale development of genome sequencing technology, the sequencing results have shown that ABC transporter genes are highly conserved in many insects (Figure 2). Aside from some important discoveries on the function of some ABC transporters in insects, however, knowledge on the role and function of these proteins is still limited. ABC transporters of numerous important agricultural pests and model insects, such as *D. melanogaster*, *B. mori*, *Helicoverpa armigera* and *Plutella xylostella*, have been reported (Table 1).

### 3.1. The ABCA Subfamily

According to the distinctive conserved traits, ABCA subfamily proteins can be divided into full transporters (FTs) and half transporters (HTs); the number of insect *ABCA* genes differs among different species (3–15). *ABCA* genes that encode only FTs are present in *T. castaneum*, *Anopheles gambiae*, *D. melanogaster*, *Apis mellifera*, *Daphnia pulex*, *Tetranychus urticae* and *Bemisia tabaci* [11,58]. Two FTs and five HTs have been found in one analysis of the *B. mori* genome [51], whereas another showed two FTs, one HT and three incomplete ABC sequences (These sequences were derived from a sequenced and assembled genome, but the genomic scaffold is incomplete.) [50]. In mammals, ABCA transporters function in controlling cellular lipid transport [65], but little is known about their specific function in insects. Silencing of *TcABCA-9A* or *TcABCA-9B* in *T. castaneum* by injection of dsRNA leads to developmental defects in the wings and elytra and to about 30% mortality at the adult eclosion stage [20]. This silencing study provides a direction for the functional analysis of insect ABCA subfamily genes. The flight capability of insects is related to lipid metabolism, the biosynthesis of various lipid classes, and the total lipids and triglycerides provide energy for flight [66,67]. As in mammals, ABCA proteins may function in lipid transport in insects, so the developmental defects in wings and elytra are likely due to loss of lipid transport. In addition, ABCA2 is also associated with insect resistance to Bt toxin [68].

### 3.2. The ABCB Subfamily

ABCB subfamily transporters also contain FTs and HTs. In the genome of *T. castaneum*, *An. gambiae*, *D. melanogaster*, *B. mori* and *A. mellifera*, the number of FTs are 2, 2, 4, 5 and 1, and the number of HTs are 4, 3, 4, 4 and 4, respectively. *A. mellifera* also contains two incomplete ABCB sequences. Similarly, two genes encoding FTs, and five and two genes encoding HTs were found in the *D. pulex* and *T. urticae* genomes, respectively. The function of the ortholog of the human half transporter *Hs*ABCB7 in *D. melanogaster* was studied by RNA interference and found to play a key role in cellular iron homeostasis [69]. *CG4225*, homologous to human *ABCB6* in *D. melanogaster*, is also known as *DmHMT-1*, which is responsible for resistance against cadmium [70]. Another ABCB transporter gene, *CG7955* in *D. melanogaster*, is correlated with chill coma stress resistance [71]. The perineurium in lepidopterans serves as a diffusion barrier for polar cardenolides and provides an active barrier for non-polar cardenolides, and the P-glycoprotein-like transporter mediates the efflux of cardenolides in the nerve cord, thereby preventing interaction of these toxins with the susceptible target site in Na^+^/K^+^-ATPase [72]. 

### 3.3. The ABCC Subfamily

The functions of ABCC subfamily in mammals mainly include ion transport, signal transduction, and toxin secretion [1]. The ABCC transporters in insect may have similar functions. Usually, insect genomes contain 9–16 *ABCC* genes, with the most found to date in *T. urticae*, followed by *T. castaneum* and *Chrysomela populi*. There are 35 genes encoding ABCC transporters in *T. castaneum*, and expansion of *TcABCC* gene mainly occurs on chromosome 5 and affects genes of the “short” MRP group [20]. The ABCC subfamily of *B. mori* can be divided into two groups, one of which has very high similarity with the ABCB subfamily [50]. The *B. mori* ABCC subfamily consists of five FTs, seven HTs and three incomplete ABC sequences. The ABCC transporters of most insects with known genomes are also composed of FTs and HTs, except for *D. melanogaster*; its 14 ABCC transporters are FTs. Some ABCC transporters also contain an additional N-terminal TMD (TMD0) [46] and have been found in the genome of *D. pulex*, *An. gambiae*, *D. melanogaster* and *T. castaneum* [20,47,48]. 

### 3.4. The ABCD, ABCE and ABCF Subfamilies

The ABCD subfamily is composed of highly conserved HTs, located on the peroxisome membrane, that form heterodimers and mediate the transport of acyl coenzyme A esters in organisms [73,74]. Most insects with sequenced genomes have two *ABCD* genes, but a few insects, such as *Danaus plexippus* and *P. xylostella*, contain three [59,60]. Because of the high sequence similarity between insects and other eukaryotes, including humans, they may be involved in similar peroxisomal pathways [11].

Compared with other ABC transporters, both ABCE and ABCF proteins have no TMDs, and each member contains a pair of adjacent NBDs [75]. Due to their lack of transmembrane structure, proteins in these two subfamilies do not have a translocation function [76]. Almost all insects, and even most eukaryotes, contain only one *ABCE* gene, which is highly conserved [50]. The ABCF subfamily is also highly conserved. Similar to human ABCF proteins, most of the known insect genomes have three ABCF proteins. They are divided into three distinct groups by phylogenetic analysis, and the genes in each group is highly similar [50]. ABCE and ABCF proteins are universally found in eukaryotes and are important in ribosome biogenesis, translation control and mRNA output [76,77,78]. *ABCE* is annotated as RNase L (Rli1) inhibitor in eukaryotes and may have a general function in innate immunity [79]. Injection of *T. castaneum* with either *TcABCE-3A*- or *TcABCF-2A*-specific dsRNA leads to a lethal phenotype [20].

### 3.5. The ABCG Subfamily

ABCG transporters are typical HTs, which must form homo- or heterodimers to fulfill their transport function. Compared with other half-transporter proteins, ABCG transporters have a reverse domain structure; its TMD is connected to the C-terminal region of NBD [14]. Some ABCG proteins can form dimers with different partners, thus increasing the repertoire of substrates they can transport [11]. The number of *ABCG* genes in an insect species ranges from 2 to 24. The white proteins of *D. melanogaster* can form dimers with one of the other two HTs named Brown and Scarlet and play a crucial role in the cellular uptake of pigment precursors in the eye [80]. An ortholog of the *white* gene of *D. melanogaster* is present in all known insect genomes, but the *brown* and *scarlet* genes are only present in some insects [11]. A study of the *white* ortholog of *T. castaneum* found that the transport precursor of a pigment across the membrane of Malpighian tubule cells is ABCG-dependent [20]. White is also necessary for the transport of uric acid in the larval epidermis of *B. mori*. The ABCG HTs homologous to brown in aphids, lepidopteran and hymenopteran species can form dimers with white to participate in the transport of urate [81]. In the *B. mori* genome, *white* and *scarlet* are juxtaposed in a head-to-tail orientation, which indicates that the origin of *white* and *scarlet* resulted from the tandem duplication of an ancestral gene. The same arrangement was also found in other insect species that lack a functional ortholog of the *brown* gene [19]. 

### 3.6. The ABCH Subfamily

ABCH transporters were first found in *D. melanogaster*, then in other insects; they have not been found in mammals, plants, fungi and *C. elegans* [1,82,83]. However, this type of ABCH protein was reported in zebrafish [84], but reports of the gene in other teleost fishes have not been consistent [85,86]. Four *ABCH* genes were identified in the *Ptychadena nana* genome [87]. Similar to the ABCG transporter, ABCH proteins are also HTs and have the reverse domain organization with the NBD connecting to the N-terminal side of the TMD [14]. Three ABCH proteins exist in most of the known insect genomes. Although this protein is very similar to the ABCG transporter, little is known about its function at present. Silencing of the *ABCH1* gene of *P. xylostella* with a high dose of dsRNA results in lethal larval and pupal phenotypes [59]; thus *ABCH1* may be an excellent target for pest control. Injection of dsRNA of *TcABCH-9C* causes dehydration and death of *T. castaneum* larvae [20]. ABCH-9C may be involved in the formation of the protective lipid barrier on the cuticle surface of *Locusta migratoria* [88,89] and *D. melanogaster* [89].

## 4. ABC Transporters in Xenobiotic Detoxification by Insects

### 4.1. Insecticide Transport and Detoxification 

Generally, insect resistance to xenobiotics is due to point mutations at target sites, or the effective metabolism or sequestration of toxic substances [90,91]. The emergence of insecticide resistance in insects is mainly related to the decreased sensitivity of target sites to insecticides and changes in the expression or properties of metabolic detoxification genes [92,93]. Several members of the insect ABC transporter superfamily play a very important role in pesticide resistance by inhibiting the accumulation of intracellular pesticides and their metabolites [94]. 

Xenobiotic transcription factors (XTFs) are also important in regulating the expression of genes that encode proteins involved in detoxification. Cap’n’collar (Cnc) transcription factors are members of the XTF superfamily [95,96]. In the red flour beetle (*T. castaneum*), the Cap’n’collar C isoform (CncC) is involved in all three phases of insecticide detoxification and regulates the expression of *ABCA-UB*, *ABCA-1A* and *ABCA-1AL*. RNAi and an insecticide bioassay showed that these *ABCA* genes contributed to the susceptibility of *T. castaneum* to pyrethroid [97]. This study provided a clearer understanding of the role of ABC transporter and its upstream regulatory pathway in insecticide detoxification.

Among ABC transporters family, the ABCB subfamily mostly represents the P-gps and multiple drug resistance (Mdr) proteins. P-gps are ATP-dependent efflux pumps, which are closely related to the transmembrane transport of substances. Some of these proteins are associated with multidrug resistance [98]. When RNAi-mediated gene silencing was used to analyze the potential function of P-gp in insecticide efflux in *Aedes aegypti*, temephos toxicity significantly increased (57%) in the *P-gp*-silenced mutant [99]. Jin et al. reported that inhibition of P-gp increased the susceptibility of *H. armigera* to abamectin and indoxacarb [62]. Previous studies by Aurade et al. have also shown that P-gp is associated with resistance of *H. armigera* to insecticides [100]. An increase in P-gp expression in *D. melanogaster* is directly related to abamectin resistance, and the level of P-gp in blood-brain barrier of resistant *D. melanogaster* is significantly higher than in the susceptible control [101,102]. In-depth analysis showed that P-gp content is regulated by epidermal growth factor receptor (EGFR) and protein kinase B (Akt, PKB) pathways in *D. melanogaster* [101]. Over-expression of *ABCB4* through gene amplification has been detected in pyrethroid-resistant *Ae. aegypti* [21]. ABCB transporter subfamily members Mdr49, Mdr50 and Mdr65 are associated with DDT resistance in 91-R strain of *D. melanogaster*. Among them, the resistance conferred by Mdr49 was related to its splice-form variant and amino acid residue changes [103,104]. *D. melanogaster* with a *Mdr65*-gene knockout via CRISPR/cas9 had increased susceptibility to all neuroactive insecticides tested. Deficiency crosses, synergism with the ABC inhibitor verapamil and accumulation of pesticides in the *Mdr65*-knockout individuals further confirmed that Mdr65 plays an important role in pesticide transport [105]. Tissue-specific RNAi of *Mdr65* also confirmed the role of the gene in reduced toxicity of multiple insecticides in *D. melanogaster* [106]. 

Many ABCC transporters can specifically transport a variety of drugs, so they are also called multidrug resistance proteins (MRPs). *Trn*MRP1 and *Trn*MRP4 are highly expressed in the Malpighian tubules in the lepidopteran *Trichoplusia ni* and may be involved in excreting metabolic wastes or ingested xenobiotics [107]. Microarray studies of adult *B. tabaci* showed that an ABC transporter of subfamily G is upregulated when exposed to thiamethoxam [108,109]. Compared with expression in susceptible strains, the expression of A, C, G, H and F subfamily members of ABC transporters was higher in chlorpyrifos- and fluronitrile-resistant strains of *P. xylostella* (diamondback moth), suggesting that these ABC transporters may be related to the transport or detoxification of different pesticides [110,111]. The expression of ABC genes *HaOG200303* from subfamily C, *HaOG200310*, *HaOG200353* and *HaOG200354* from subfamily G and *HaOG200341* from subfamily H were significantly upregulated in *H. armigera* when treated with both lambda-cyhalothrin and indoxacarb [62]. However, whether the high expression of ABC transporter after insecticide induction is related to the detoxification metabolism of insects needs to be confirmed by further studies. *PhABCC4* of *Pediculus humanus humanus* expressed in Xenopus oocytes functioned in ivermectin efflux, suggesting that *Ph*ABCC4 might be involved in ivermectin transport [112].

### 4.2. Detoxification of Plant Secondary Metabolites

Plants can release a variety of toxic secondary metabolites to deter the feeding of herbivorous insects, and insects have evolved corresponding countermeasures via natural selection, specialization, sequestration during the long-term interactions between plants and insects [113]. As part of these countermeasures, members of ABC transporter family play a crucial role in overcoming multiple chemical plant defenses. A gene knockout and bioassay in *D. melanogaster* indicates that Mdr is involved in the detoxification of plant secondary metabolite cardenolides [114]. A study of the ABC transporter *Cp*MRP (ABC subfamily C) of *C. populi* revealed that *Cp*MRP acts as a pacemaker, transporting specific metabolites from the hemolymph to defensive secretions, and eventually excreting them from the body. Silencing of *CpMRP* makes larvae defenseless, indicating *Cp*MRP plays a key role in secretion [115]. Homologous sequences of *Cp*MRP were also identified in the defensive glands of two related leaf beetle species, suggesting that this ABC transporter is the key component of insect resistance to noxious phytochemicals. 

The expression level of *ABC* genes was studied in tissues of 5th instar larvae of *H. armigera* after they were fed an artificial diet supplemented with various plant secondary metabolites. Taxol induced the expression of *ABCE1* in Malpighian tubules, upregulated the expression of *ABCB1* in the gut and expression of *ABCB1*, *ABCC2* and *ABCC6* in the rest of the body [61]. ABCB1 has also been shown to be associated with paclitaxel resistance of human cancer cells [116]. The expression of *HaABCG11* was upregulated after larvae were fed a diet supplemented with nicotine compared to the control samples, and *HaABCB3* expression was very high in the gut of *H. armigera* larvae after they fed on a diet containing nicotine or tomatine [61]. Similarly, *ABCB3* was upregulated in *Manduca sexta* larvae that fed on *Datura* and *Solanum*. This plant-specific expression pattern may be related to the adaptability of insect to these plants [117]. The function of ABCB3 in insects is not completely understood, but it most likely is important in a defense response of insects to plant secondary metabolites. 

## 5. ABC Transporter Roles in Bt Insecticidal Activity

The ABC transporters that act as Bt-toxin receptor belong to subfamilies A, B, C and D (Table 2). With the long-term cultivation of Bt cotton, field resistance to Cry2Ab toxin evolved in *H. armigera*. The resistance of two *H. armigera* strains to Cry2Ab was an independent evolutionary event involving different deletion mutations, which were located in different exons of the same *ABCA2* gene [68]. A homologous deletion in *HaABCA2* was also detected in resistant lines of *H. punctigera* Cry2Ab. A similar situation was also found in resistant lines of *Pectinophora gossypiella*; a loss of *PgABCA2* exon 6 was caused by alternative splicing in resistant larvae of laboratory-selected strains in Arizona and field-selected strains in India [118]. In addition, insect ABCA2 was expressed in the midgut where the Cry2Ab toxin binds [68,119]. A bioassay of two strains of *H. armigera* with CRISPR/cas9 knockout of *HaABCA2* showed that *Ha*ABCA2 plays an important role in mediating resistance to Cry2Aa and Cry2Ab [120].

A *Chrysomela tremuela* strain survived and reproduced on transgenic poplar trees that expressed a high level of Cry3Aa Bt toxin, and its Cry3Aa resistance was an autosomal recessive trait. Candidate resistance genes were analyzed using midgut transcriptome of larvae, demonstrating that a mutation of the *ABCB* homolog of *P-gp* was closely linked to the resistance of Cry3Aa, and named *CtABCB1*. A 4-bp deletion in *CtABCB1* introduced a frame shift with a premature stop codon, resulting in the loss of transporter signature motifs 1 (TpM1) and transmembrane domain 2 (TMD2) [130]. A *CtABCB1* homologous gene in western corn rootworm (*Diabrotica virgifera virgifera*) was also shown to be genetically linked to Cry3Bb1 resistance [131]. Perhaps the insect ABCB1 transporter is a receptor specific for Cry3 toxin, and structural changes in this transporter may be associated with Cry3 toxin resistance of the insect.

Some ABCC transporters are a functional receptor for more than one Cry1A toxin. The binding of Cry1Ac toxin to ABCC2 on membrane vesicles can lead to membrane perforation. Bretschneider et al. studied the relationship between the ABCC2 of *Heliothis virescens* and cytotoxicity of three Cry1A toxins (Cry1Aa, Cry1Ab, Cry1Ac) in *Spodoptera frugiperda* Sf9 cells and found that ABCC2 is the central target of Cry1A toxin action [132]. Heterologous expression in *D. melanogaster* has been used to validate the involvement of ABCC2 in Cry1Ac toxin binding [133]. An inactivating mutation, such as mis-splicing of *ABCC2* or a mutation causing a single amino acid change, could reduce binding of Cry1Ac toxin, conferring high levels of Bt resistance in the target pest [25,124,134]. In a Cry1Ac-resistant strain of *P. xylostella* with a 30-bp deletion in exon 20, was expected to cause the removal of the 12th transmembrane (TM) domain and carboxyl terminal of TM12 that is located outside the cell. The mutation may result in the lack of function of a core ATP-binding loop [126]. In addition, an ABCC transporter is also the receptor of Cry1Ca and Cry1Fa toxins [127,129]. A cytotoxicity assay showed that the binding affinity of ABCC transporters to Cry toxin was largely linked to the susceptibility of receptor-expressing cells to Cry toxin and that the extracellular loop (ECL) structures determine the specificity of ABCC to Cry toxins [135]. 

Bt resistance mediated by ABCC transporters involves not only different forms of gene mutations, but also the regulation of *ABCC* gene expression. The study of Bt resistance in hybrid larvae of *B. mori* revealed a *trans*-regulatory mechanism involved in the allele-specific expression of *ABCC2* in response to Cry1Ab toxin, which may play an important role in insect Bt resistance [122]. The binding of Cry1Ac toxin to ABCC transporters that causes midgut membrane perforation is regulated by a constitutively and transcriptionally activated upstream gene (*MAP4K4*) in the MAPK signaling pathway [125]. In addition, Forkhead box protein A (FOXA) upregulates expression of *ABCC2* and *ABCC3* genes in Sf9 cells [128]. ABCC2 and ABCC3 are important receptors of Cry1Ac toxin, and their expression level has a significant influence on insect Bt resistance; thus, FOXA may be involved in the regulation of insect resistance to Cry1Ac toxin.

*ABCG1* (*white*) gene is one of the most widely studied members of the ABCG protein subfamily. The white protein in *P. xylostella* is located on the cell membrane, and the expression of *Pxwhite* gene in the midgut of a Bt-resistant population is significantly lower than in a susceptible population. After RNAi silencing of the midgut *Pxwhite* gene, the sensitivity of the larvae to Bt-Cry1Ac toxin significantly decreased [136]. Genetic linkage analysis confirmed that the decrease was closely linked to the Bt-Cry1Ac resistance of the moth. Downregulated expression of ABCG subfamily genes in *Ostrinia furnacalis* is also related to its resistance to Cry1Ab and Cry1Ac toxins [123].

## 6. Discussion

Functional study of insect ABC transporters and their role in resistance to chemical insecticides and Bt toxins has shown that several ABC transporters are involved in toxin resistance. However, more progress is needed to fully understand the functions and detailed mechanisms of action. Different ABC transporters may recognize different chemicals or Bt toxins, and their TMDs have distinct binding sites that recognize substrates with highly diverse chemical properties [137]. ABCA2 is the receptor of Cry2Ab toxin [68,118,119], ABCB1 is the receptor of Cry3 toxin [130,131], and members of the ABCC family are receptors of Cry1 toxin [25,124,132,134]. Thus, different pesticides, toxic secondary metabolites of plants and Bt toxins may be recognized and transported by different ABC transporters. The strategies and methods that are used to study human ABC transporters involved in drug transport can also be used to investigate ABC transporter functions and mechanisms involved in insect resistance. The sequencing of insect genomes provides unparalleled advantages over traditional methods for revealing the molecular basis and mechanisms of insect resistance to pesticides and Bt toxins, and insect genomics approaches will become the most important methods to analyze insect resistance mechanism.

ABC transporters are the main receptors for most Bt toxins, so it is not surprising that Bt resistance in pests is commonly related to a mutation in an ABC transporter. Upstream regulatory factors such as MAP4K4 and FOXA can also change gene expression for ABC transporters, thereby altering susceptibility of pests to Bt toxins [125,128]. The regulatory pathways and corresponding mechanisms that affect the expression of ABC transporter need to be further elucidated. If the regulation mechanism of the expression of ABC transporter can be clearly understood, more efficient and convenient measures can be developed for Bt resistance control. Insects that are resistant to chemical pesticides, plant toxic secondary metabolites and Bt toxins may also be subject to a fitness cost. Any such physiological cost correlated with resistance may also provide a target for pest control.

Plant-mediated RNA interference provides a new method and model for pest control in the field. The dsRNA fragment targeting the *β-actin* gene in Colorado potato beetle was transferred to potato chloroplasts, and the transgenic plants were lethal to Colorado potato beetle larvae [138]. The use of dsRNA to knockdown the *acetylcholinesterase* (*AChE*) gene involved in neuronal transmission and the *ecdysone receptor* (*EcR*) gene involved in transcriptional activation of development in transgenic plants can effectively control whitefly populations [139]. Because insect resistance to chemical pesticides is related to the upregulation of certain ABC transporters, silencing the genes for these ABC transporters is expected to make the pest more susceptible to the pesticide. In addition, the development of inhibitors specific for a particular ABC transporter might enhance the susceptibility of the target insect. Since ABC transporters can differ significantly among various species, the inhibitor must be highly specific for the transporter involved in the target(s) species and not adversely affect other insects or organisms.

With the deepening of our knowledge on the structure and physiological function of insect ABC transporters, the mechanism of action of these transporters in pesticide resistance, Bt insecticidal activity and other physiological processes will become clearer, and new technologies will be developed for the effective control of insect pests.

## Figures and Tables

**Table 1:** Distribution of genes among ABC transporter subfamilies for different arthropods and *Homo sapiens*.

Organisms	A	B	C	D	E	F	G	H	Total	References
*Homo sapiens*	12	11	12	4	1	3	5	0	48	[1]
*Drosophila melanogaster*	10	8	14	2	1	3	15	3	56	[1]
*Anopheles gambiae*	9	5	13	2	1	3	16	3	52	[47]
*Daphnia pulex*	4	7	7	3	1	4	24	15	65	[48]
*Pediculus humanus humanus*	2	6	5	2	1	3	13	6	40 ^a^	[49]
*Apis melifera*	3	5	9	2	1	3	15	3	41	[50]
*Bombyx mori*	7	9	11	2	1	3	16	2	51 ^b^	[50,51,52]
*Tribolium castaneum*	10	6	35	2	1	3	13	3	73	[20]
*Tetranychus urticae*	9	4	39	2	1	3	23	22	103	[53]
*Chrysomela populi*	5	8	29	2	1	3	14	3	65	[54]
*Lygus hesperus*	11	6	12	2	1	3	19	11	65	[55]
*Lepeophtheirus salmonis*	3	4	11	3	1	4	2	5	33	[56]
*Laodelphax striatellux*	2	6	5	2	1	2	14	8	40	[57]
*Bemisia tabaci*	8	3	6	2	1	3	23	9	55	[58]
*Plutella xylostella*	15	14	21	3	1	3	19	6	82	[59,60]
*Manduca sexta*	7	9	11	2	1	3	13	3	52	[60]
*Danaus plexippus*	8	16	12	3	1	3	16	3	62	[60]
*Heliconius melpomene*	10	11	15	2	1	3	17	3	62	[60]
*Helicoverpa armigera*	7	11	11	2	1	3	17	2	54	[52,61,62]
*Helicoverpa zea*	7	11	11	2	1	3	17	2	54	[52]
*Acyrthosiphon pisum*	11	9	16	2	1	4	19	9	71	[63,64]

^a^ Includes two uncharacterized ABC transporters. ^b^ The number of *B. mori* ABC transporter genes in the different subfamilies is mainly based on the latest report [52], but were reported previously [50].

**Table 2:** Bt toxins and their possible corresponding ABC transporters as receptors in insects.

Bt toxin	Receptor	Target pest	Reference
Cry1Aa	ABCC2	*Bombyx mori*	[121]
Cry1Ab	ABCC2	*Bombyx mori*	[122]
	ABCG1	*Ostrinia furnacalis*	[123]
Cry1Ac	ABCC2	*Helicoverpa armigera*	[25]
	ABCC2	*Heliothis virescens*	[124]
	ABCG1	*Ostrinia furnacalis*	[123]
	ABCG1	*Plutella xylostella*	[125]
	ABCC2	*Plutella xylostella*	[126]
	ABCC2	*Spodoptera exigua*	[127]
	ABCC3	*Spodoptera frugiperda*	[128]
Cry1Ca	ABCC2	*Spodoptera exigua*	[127]
Cry1Fa	ABCC2	*Ostrinia nubilalis*	[129]
Cry2Aa	ABCA2	*Helicoverpa armigera*	[120]
Cry2Ab	ABCA2	*Helicoverpa armigera*	[68]
	ABCA2	*Helicoverpa punctigera*	[68]
	ABCA2	*Pectinophora gossypiella*	[118]
Cry3Aa	ABCB1	*Chrysomela tremuela*	[130]
Cry3Bb1	ABCB1	*Diabrotica virgifera virgifera*	[131]

## Supplementary Materials

Supplementary materials can be found at https://www.mdpi.com/1422-0067/20/11/2829/s1.
